# Carboxyamidotriazole combined with IDO1-Kyn-AhR pathway inhibitors profoundly enhances cancer immunotherapy

**DOI:** 10.1186/s40425-019-0725-7

**Published:** 2019-09-11

**Authors:** Jing Shi, Chen Chen, Rui Ju, Qingzhu Wang, Juan Li, Lei Guo, Caiying Ye, Dechang Zhang

**Affiliations:** Department of Pharmacology, Institute of Basic Medical Sciences, Chinese Academy of Medical Sciences and School of Basic Medicine, Peking Union Medical College, Beijing, China

**Keywords:** CAI, PD-1, IFN-, IDO1, AhR

## Abstract

**Background:**

Cancer immunotherapy has generated significant excitement, mainly as a result of the development of immune checkpoint inhibitors. The blockade of PD-1 or its ligand with antibodies has resulted in impressive clinical efficacy. However, a subset of patients does not respond to biologic therapeutics, and another subset suffers from severe immune-related adverse events in certain cases. The modulation of the immune system with small molecules might yield surprising benefits.

**Methods:**

CD8^+^ cells were obtained through a magnetic cell sorting system (MACS), and their capabilities for IFN-γ release and PD-1 expression were analyzed. The in vitro effects of drugs were studied in a coculture system of tumor cells and activated CD8^+^ cells. We further isolated the primary tumor cells in tumor-bearing mice treated with CAI, DMF, 1-MT or a combination (CAI and DMF/CAI and 1-MT) and analyzed the percentages of CD8^+^ T cells and PD-1^+^CD8^+^ T cells among TILs. The selective anti-tumor immune reactions of the two drug combinations were confirmed in a coculture system consisting of B16-OVA cells and OVA-specific CTLs derived from OT-1 transgenic mice. The anti-tumor effects of the single drugs or combined therapies were assessed according to their capability to slow tumor growth and extend the life span of tumor-bearing mice, and they were compared with the effects of PD-1 antibody.

**Results:**

CAI increased IFN-γ release from activated T cells, which might strengthen the anti-proliferative and anti-metastatic effects on cancer cells. However, CAI also stimulated IDO1-Kyn metabolic circuitry in the tumor microenvironment and facilitated tumor cell immune evasion. Combining CAI with 1-MT or DMF disrupted PD-1 expression and promoted IFN-γ production in CD8^+^ T cells, and it also increased T lymphocyte infiltration in the tumor microenvironment, inhibited tumor growth and prolonged the life spans of tumor-bearing mice.

**Conclusion:**

Inhibitors of the IDO1-Kyn-AhR pathway could abolish the negative effects of CAI on CD8^+^ T cells and result in complementary and beneficial anti-tumor immune effects. The combination of CAI with 1-MT or DMF greatly augmented the ability of CD8^+^ T cells to kill malignant cells and showed a strong anti-cancer capability that was superior to that of either of the single agents was is comparable with that of anti-PD-1 antibody. The combinations of small molecules utilized in this study may serve as valuable new immunotherapy strategies for cancer treatment.

**Electronic supplementary material:**

The online version of this article (10.1186/s40425-019-0725-7) contains supplementary material, which is available to authorized users.

## Introduction

Cancer immunotherapy harnesses the immune system to fight cancer by either stimulating the functions of specific components of the immune system or counteracting the signals that protect tumor cells from immune defense [1]. As one of the most important drug discoveries, specific inhibitors against programmed death 1 (PD-1) or its main ligand PD-L1 have achieved prominent clinical success [2, 3]. PD-1 is an inhibitory receptor expressed on T cells, and PD-L1, the ligand of PD-1, is upregulated by interferon γ (IFN-γ) and other cytokines produced after T cell activation [4]. The binding of PD-L1 to PD-1 promotes T cell apoptosis, anergy, and functional exhaustion and serves as an important mechanism of cancer immune evasion [5]. Therefore, antibodies that block PD-1 or PD-L1 provide a new benchmark for cancer immunotherapy, leading the way for developing new immunotherapeutic approaches [6].

Carboxyamidotriazole (CAI) exposure has been demonstrated to inhibit the growth of a variety of cancer cell lines [7–10]. Despite the disease stabilization and improvement in performance status observed in patients with refractory cancers after CAI treatment [8, 11], CAI has failed to provide clinical benefit or improvement when used with other therapeutic modalities [12, 13]. Previously, we found that CAI results in anti-inflammatory activity in addition to its anti-tumor effect and is capable of regulating the secretion of a variety of cytokines [14, 15]. Recently, we focused on the increased level of IFN-γ production in T cells after CAI treatment. IFN-γ is a multipotent cytokine with cytostatic/cytotoxic activity during the cell-mediated adaptive immune response, which is produced mainly by cytotoxic T lymphocytes (CTLs) and NK cells. IFN-γ has also been reported to upregulate immunosuppressive molecules such as PD-L1 and IDO1, thus promoting tumor immune escape [4, 16]. Considering its IFN-γ-stimulating effects, CAI might play a unique role in anti-tumor immunity. We speculate that the mild anti-cancer effects of CAI might be due to some adverse factors that can impair its action. A prototypical integrative modifier, indoleamine 2,3-dioxygenase-1 (IDO1), which bridges inflammation, vascularization, and immune escape and can be enhanced by IFN-γ, has become our research focus.

IDO1 is the initial rate-limiting enzyme in tryptophan (Trp) catabolism within the Kyn pathway. The overexpression of IDO1 may lead to tryptophan depletion and metabolite (Kyn, kynurenic acid, 3-hydroxy-kynurenine, etc.) accumulation, which can actively suppress T-cell functioning [17]. In addition, Kyn and Kyn derivatives can bind the aryl hydrocarbon receptor (AhR) [18], which has been shown to impair the proliferation and function of various immune effectors, including CD8^+^ T lymphocytes, and provide tumor cells with a means to evade anticancer immunosurveillance [19].

In the present study, we provide evidence that the effects that hamper the in vivo anti-tumor capability of CAI might occur through the IDO-Kyn-AhR cascade. 1-methyl-L-tryptophan (1-MT), a tryptophan derivative that disrupts IDO1 signaling [20], or 3′,4′-dimethoxyflavone (DMF), an antagonist of AhR that inhibits the Kyn-AhR pathway [21] were both used in combination with CAI. The two combinations (CAI + 1-MT and CAI + DMF) greatly enhanced PD-1 blockade in CD8^+^ T cells, enhancing the anti-cancer capacity of the anti-PD-1 antibody. This provides a valuable immunotherapy strategy for cancer by using low-cost small molecule drug combinations with a favorable toxicity profiles (Additional file 1: Figure S1).

## Materials and methods

### Cell lines and reagents

Mouse tumor cell lines B16 (melanoma), OVA-B16 (melanoma), C26 (colon cancer) and 4 T1 (breast cancer) were purchased from the China Center for Type Culture Collection (Beijing, China) and cultured in RPMI 1640 (Thermo Fisher, MA, USA) with 10% fetal bovine serum (FBS) (Gibco, MA, USA), with the exception of 4 T1 cells, which were grown in DMEM medium (Gibco, MA, USA) with 10% FBS.

Carboxyamidotriazole was synthesized by the Institute of Materia Medica, Chinese Academy of Medical Sciences (Beijing, China). Polyethylene glycol 400 (PEG400) was obtained from Sinopharm Chemical Reagent Beijing (Beijing, China). 1-Methyl-L-tryptophan, 3′, 4′-dimethoxyflavone and L-kynurenine sulfate salt were purchased from Sigma-Aldrich (Saint Louis, USA).

### CD8^+^ T cell sorting

CD8^+^ T cells were isolated from the spleens of BALB/c mice using a negative magnetic cell separation kit (MACS, Mouse Naive CD8^+^ T Cell Isolation Kit, Miltenyi Biotec). The cell purity (above 95%) was confirmed by flow cytometry using an anti-mouse CD8 antibody (eBioscience, CA, USA). The isolated CD8^+^ T cells were cultured in RPMI 1640 medium containing 10% FBS and 10 ng/ml IL-2 (Peprotech, NJ, USA) and activated with 1 mg/ml anti-mouse CD3/CD28 microbeads (Thermo Fisher, MA, USA).

Human naive CD8^+^ T cells were isolated from human peripheral blood monocytes (PBMCs). Briefly, human blood samples were collected from 12 healthy donors, and then the samples were subjected to density gradient centrifugation to obtain the PBMCs. CD8^+^ T cells were isolated using positive MACS (Human Naive CD8^+^ T Cell Isolation Kit, Miltenyi Biotec). The cell purity was confirmed with the same method described above, and the same cell culture conditions were used, except that the antibody, recombinant protein and microbeads used were human-specific.

### Animal experiments and treatment protocol

C57BL/6, BALB/c or RAG1 KO mice were subcutaneously injected with appropriate amounts of the indicated tumor cells (B16, C26, 4 T1 or B16-OVA) in the right flank. Three days after inoculation or after the tumor size reached 5 × 5 mm, the mice were randomized into different groups (*n* = 6~10). Then, the mice in each group were treated with the following drugs separately for the indicated time: CAI (intragastric injection of 20 mg/kg/day), anti-IFN-γ neutralizing antibody (250 mg per mouse),1-MT (5 mg/ml in drinking water, 3–4 ml/mouse/day), CAI + 1-MT (the same as that used for monotherapy), DMF (intragastric injection of 10 mg/kg once every 2 days), CAI + DMF (the same as that used for monotherapy), and anti-PD-1 neutralizing antibody (250 μg per mouse). Kyn was administered by intratumoral injection (20 mg/kg/day once every 2 days). The mice in the control group received an equal volume of saline as a mock treatment. Tumor growth and the survival of the mice were recorded daily. The tumor volume was calculated according to the following formula: tumor volume = length×width^2^/2.

### Total RNA extraction and RT–PCR

Total RNA was isolated from CD8^+^ T cells using a Pure RNA Extraction Kit (BioTeke Corporation) and reverse-transcribed into cDNA with the TransScript First-Strand cDNA Synthesis Supermix (TransGen Biotech Co., Beijing, China). The primer sequences used were: IDO1, 5′-TGGCGTATGTGTGGAACCG-3′ (sense) and 5′-CTCGCAGTAGGGAACAGCAA-3′ (anti-sense); GAPDH, 5′AGGTCGGTGTGAACGGATTTG-3′ (sense) and 5′-TGTAGACCATGTAGTTGAGGTCA-3′ (anti-sense). Real-time PCR was performed using an IQ5 Real-Time System (BioRad, CA, USA). The values are the mean ± SEM of three independent experiments.

### Western blotting

CD8^+^ T cell lysate containing 40 μg of protein was subjected to SDS/PAGE, and the separated proteins were transferred onto PVDF membranes. After being blocked with 5% nonfat dry milk in Tris-buffered saline containing Tween-20, the membrane was incubated with the following primary antibodies overnight: anti-mouse IDO1 (Cell Signaling, Cat No. 86630; 1:1000), anti-mouse β-actin (Cell Signaling, Cat No. 3700; 1:1000). Subsequently, the membrane was incubated with the appropriate secondary antibody, and the immunoreactive protein bands were visualized using a chemiluminescence kit (Millipore, MA, USA) followed by ECL-based autoradiography. The Western blots are representative of at least three independent experiments.

### Cytokine release and Kyn production

Cytokine production in the supernatants was quantified by ELISA kits (BioVision, CA, USA) according to the manufacturer’s protocol. Kyn production was measured by ELISA (MYBioSource, CA, USA) according to the manufacturer’s instructions.

### Immunofluorescence

Cells cultured in the soft 90-Pa 3D fibrin gels were treated with dispase II (Roche, Swiss) for 10 min at 37 °C and then fixed with 4% paraformaldehyde, collected, embedded in paraffin, and sectioned. The sections were baked for 30 min at 60 °C, dewaxed, blocked in 2% BSA and stained with anti-AhR primary antibodies (Abcam, UK), followed by staining with Alexa 488-conjugated donkey anti-rabbit IgG secondary antibodies (Invitrogen, CA, USA). After 4,6-diamidino-2-phenylindole (DAPI) staining, the slides were mounted in Fluoromount G (Solarbio, Beijing, China) and stored at 4 °C in the dark. Images were collected by confocal microscopy.

### Preparation of single-cell suspensions from implanted mouse tumors

Mice were killed at specific time points. The tumors were dissected, washed in PBS, digested with IV collagenase (Sigma, St. Louis, USA), and then transferred to RPMI 1640 medium (Gibco, USA) supplemented with 10% FBS and incubated at 37 °C for 1 h. Then, the digested tumor tissues were dispersed into ground glass, and the tissue suspensions were filtered through a 40 μm mesh (BD Falcon, CA, USA). Red blood cell lysis buffer (eBioscience, CA, USA) was added and incubated with the samples for 5 min at room temperature. The cells were washed three times with PBS and then resuspended in PBS for experiments.

### Flow cytometry

For the flow cytometry analysis, CD8^+^ T cells were stained with APC-conjugated anti-mouse PD-1 Ab and FITC-conjugated anti-mouse IFN-γ Ab (eBioscience, CA, USA). To evaluate the tumor-infiltrating lymphocytes (TILs), a single-cell suspension from the implanted tumors was stained with the following Abs: APC-conjugated anti-mouse CD3, PE-conjugated anti-mouse CD8 and FITC-conjugated anti-mouse CD4 (eBioscience, CA, USA). Flow cytometry was performed on a BD Accuri C6 flow cytometer (BD Bioscience) and analyzed with BD Accuri C6 software.

### ChIP-qPCR assay

In brief, complete CD8^+^ T cells for the ChIP assays were prepared according to the instructions for the ChIP-IT® Express Chromatin Immunoprecipitation Kit (Active Motif, CA, USA). Every group included 5 × 10^7^ cells. Anti-mouse AhR antibody was used for chromatin immunoprecipitation (Cell Signaling, Cat No. 83200; 1:50). Control rabbit IgG was purchased from Cell Signaling. DNA was isolated and subjected to real-time PCR analysis. The following primers were used for promoter quantification: mouse PD-1 AhR 5′-GATGTGCTGACAGCCTGCTG-3′ (sense) and 5′-ATGCTCAGGGTAGCAAGACCC-3′ (anti-sense). All sequences were designed to produce amplicons that were < 200 bp. Real-time PCR amplification was carried out, and the amplification of each target gene is shown in terms of the fold enrichment compared to that of the relevant antibody control.

### Adoptive T-cell transfer

C57BL/6 J CD45.1 mice were injected subcutaneously in the abdomen with 1 × 10^5^ B16-OVA tumor cells per mouse. When the tumor size reached 5 mm in diameter, the mice were divided randomly and received one of the following treatments: vehicle, CTL (1 × 10^7^ cells/mouse once every five days three times), CTL + CAI (intratumoral injection, 20 mg/kg/day once every 2 days), CTL + DMF (intragastric injection, 10 mg/kg once every 2 days) or CTL + 1-MT (5 mg/ml in drinking water, 3–4 ml/mouse/day), or CTL + CAI + DMF, CTL + CAI + 1-MT, or anti-PD-1 neutralizing antibody (250 μg per mouse). CD45.2^+^ CTLs were isolated from the spleens of OT-1 mice and cultured with OVA peptide for 48 h. For some experiments, the mice were sacrificed 5 days after adoptive T-cell transfer to obtain the TILs.

### Statistical analysis

Data are presented as the mean ± SEM, and n represents the number of experiments or animals. The statistical significance of the differences between two groups was determined by Student’s t test or one-way ANOVA followed by Dunnett’s t-test. All statistical analyses were performed by using GraphPad Prism 6.0 software. *P*-values < 0.05 were considered statistically significant.

## Results

### CAI improves the cell killing capability of CD8^+^ T cells by increasing IFN-γ levels

**Fig. 1:**
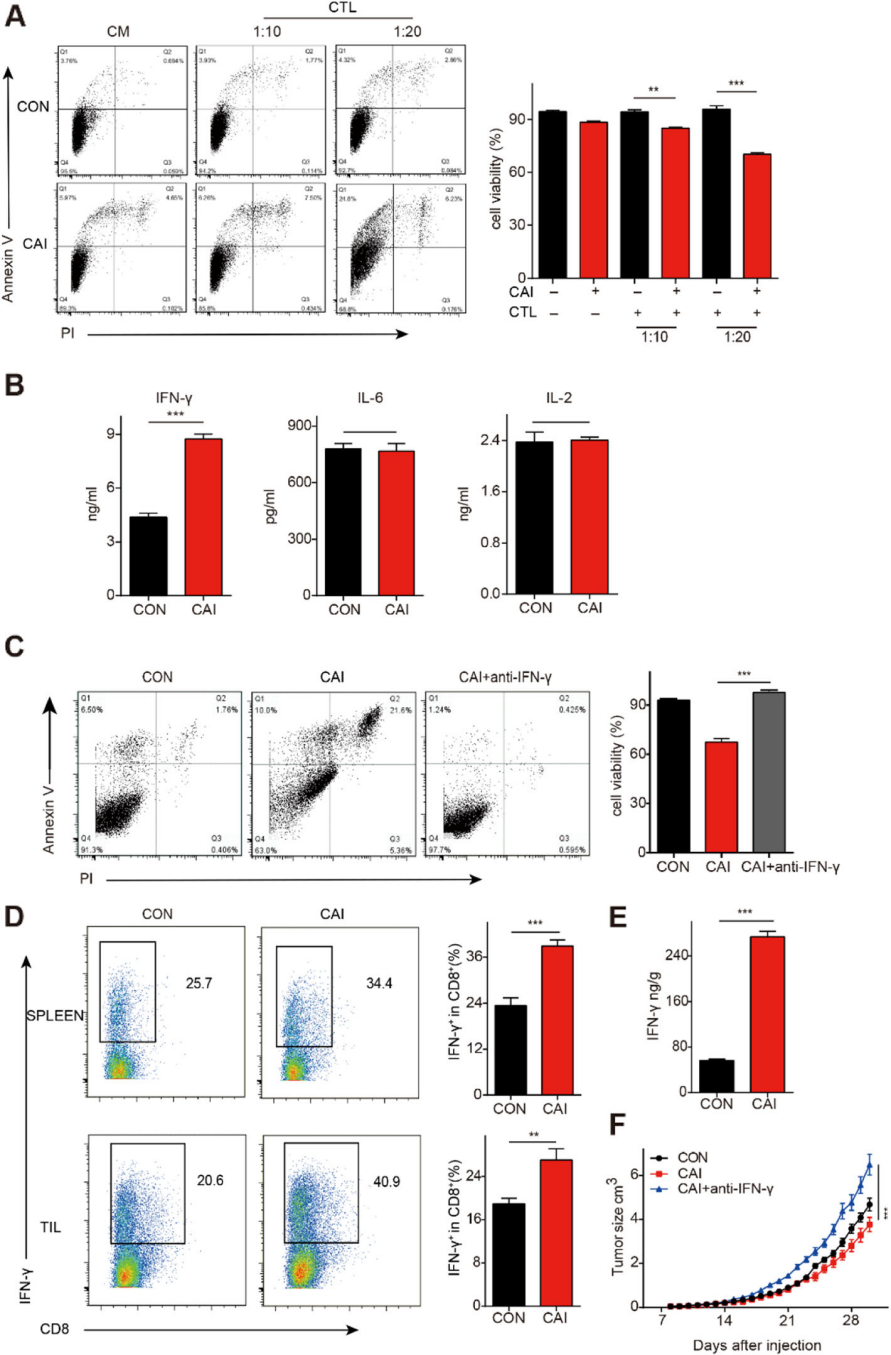
| CAI improves the cytotoxicity of CD8^+^ T cells and increases IFN-γ production. **a** B16 tumor cells and CTLs were cocultured at a ratio of 1:10 or 1:20 in the presence or absence of CAI (10 μM) for 24 h. The CTLs were preactivated with anti-CD3/CD28 beads for 48 h. The proportion of tumor cell apoptosis was determined by flow cytometry (quadrantal diagram), and the survival rate of the tumor cells in each group is shown in the bar chart. CM: culture medium (**b**) Contents of the cytokines in the supernatants of cocultured cells. **c** B16 cells were cocultured with activated CTLs at a ratio of 1:20 in the presence of vehicle (DMSO), CAI (10 μM) or IFN-γ antibody (10 mg/mL) for 24 h. The quadrantal diagrams show the proportions of tumor cell apoptosis, and the bar chart shows the survival rate of the tumor cells in each group. **d**, **e** and **f**) Mice were s.c. injected with 2 × 10^5^ B16 (*n* = 10 per group). When the average tumor size reached approximately 3 × 3 mm, the following treatments were initiated: PBS or CAI (20 mg/kg) or a combination of CAI and anti-IFN-γ antibody (250 mg/day) every 2 days for 23 days. **d** IFN-γ production in TILs and spleen was analyzed by flow cytometry. **e** Interferon content in tumor tissue was detected by ELISA. **f** Tumor growth curves. The data represent the mean ± s.e.m. *N.S.*, no significant difference; **p < 0.01, ***p < 0.001 by Student’s t test (**a**, **b**, **d** and **e**) or one-way ANOVA (**c** and **f**)

In this study, B16 melanoma tumor cells and CTLs were cocultured in the presence or absence of CAI for 24 h. CTLs exposed to CAI showed stronger cytotoxic activity against tumor cells than those not exposed to CAI, and the tumor-killing capacity was T cell number-dependent (Fig. 1a). Furthermore, when CD8^+^ T cells were pretreated with CAI for 48 h and then cocultured with tumor cells, the cytotoxicity of the CD8^+^ T cells was similar to that of CD8^+^ T cells exposed to CAI during cell coculture, indicating that CAI might promote CTL activity directly (Additional file 2: Figure S2A). The enhancement of the anti-tumor activity of CTLs by cotreatment with CAI was also observed when CTLs were cocultured with other types of tumor cells (Additional file 2: Figure S2B). Given that cytokines play critical roles in the proper establishment of anti-tumor immunity, we examined the levels of IFN-γ, IL-6 and IL-2 in both murine- and human-derived CD8^+^ T cells and in tumor cell coculture systems. IFN-γ production by CTLs was greatly enhanced by CAI (Fig. 1b and Additional file 2: Figure S2C). To gain further insight into the involvement of IFN-γ, we added IFN-γ neutralizing antibody to CAI-processed cocultured CTLs and B16 cells. The neutralizing antibody significantly counteracted the CAI-induced enhancement of the cytotoxicity of CTLs (Fig. 1c). In addition, CAI could also promote IFN-γ release from activated spleen lymphocytes and TILs in tumor-bearing mice (Fig. 1d, e, Additional file 2: Figure S2D), suggesting that there was a common phenomenon in terms of the effect of CAI on T cells. In B16 melanoma-bearing mice, CAI treatment could definitely delay tumor growth; however, the concurrent injection of anti-IFN-γ antibody and CAI not only eliminated the beneficial effect of CAI but also promoted tumor growth (Fig. 1f). These data suggest that in an environment where tumor cells and CD8^+^ T cells coexist, the enhancement of tumor anti-immunity by CAI is closely related to the release of IFN-γ.

### CAI stimulates IDO-Kyn metabolic circuitry and masks the underlying deficits of T cells via mechanisms involving Kyn-AhR activation

**Fig. 2:**
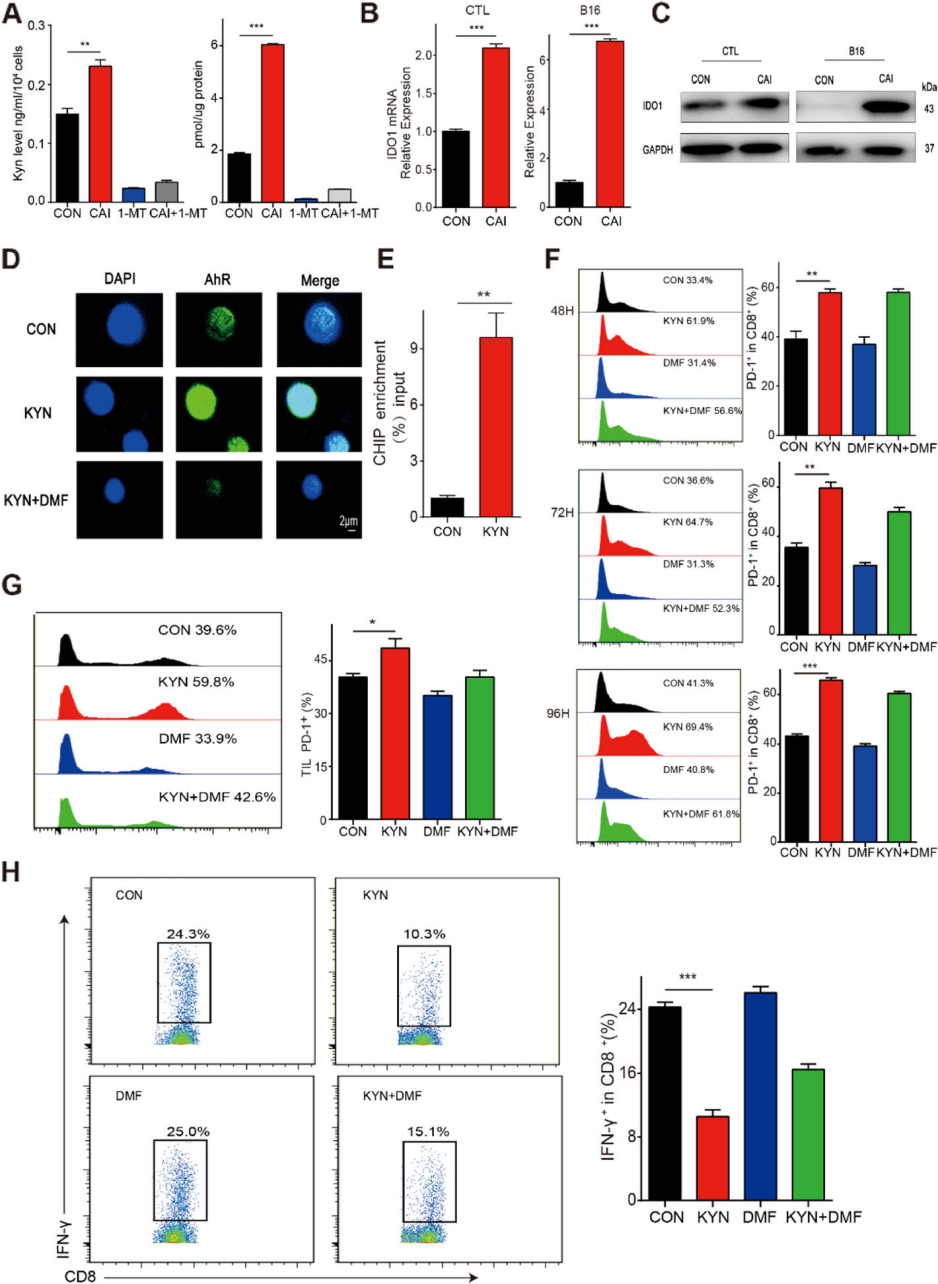
| CAI stimulation of the IDO-Kyn metabolic circuitry and the effects of the metabolite Kyn on CD8^+^ T cells. After CAI treatment (10 μM, 48 h) (**a**), the production of Kyn in the B16/T cell coculture system (left) and B16 tumor tissues (right) were determined. **b** and **c** The mRNA and protein expression of IDO1 determined by RT-PCR and Western blotting. **d** CTLs were treated with 200 mM Kyn for 2 days. The transfer of AhR from the cytosol to the nucleus determined by immunostaining assay. Bar, 2 μm. **e** ChIP-qPCR analysis of AhR-dependent PD-1 expression after Kyn treatment. The ChIP enrichment ratio relative to the control is shown. **f** CTLs were incubated with vehicle (DMSO), Kyn (200 mM) or DMF (20 μM) alone or a combination of Kyn and DMF for the indicated time spans, and the PD-1^+^ CD8^+^ T cells were analyzed by flow cytometry. Representative histograms (left) and the overall results (right) are shown. **g** B16 tumor-bearing mice received an intratumoral injection of Kyn with or without DMF treatment (10 mg/kg). Tumor-infiltrating lymphocytes (TILs) were then isolated from the tumor tissues, and the PD-1^+^ CD8^+^ T cells were analyzed by flow cytometry. Representative histogram (left) and the statistical histogram (right) are shown. **h** Intratumoral injection of Kyn reduced the proportion of IFN-γ-positive T cells in TILs isolated from B16 tumor tissues, and DMF treatment (10 mg/kg) rescued this inhibition. Representative histograms (left) and the statistical histograms (right) are shown. Data are from three independent experiments, and the error bars represent the mean ± s.e.m. *p < 0.05, **p < 0.01, ***p < 0.001 by one-way ANOVA (**a**, **g**, **f** and **h**) or Student’s t test (**b** and **e**)

To determine the reason why CAI has a weaker anti-cancer effect in vivo than expected, we tested the metabolic status of tryptophan (Trp). Intriguingly, increased Kyn concentrations were found in the supernatant of B16 cells cocultured with CD8^+^ T cells and in B16 tumor tissues from mice treated with CAI (Fig. 2a). 1-MT reduced Kyn basal levels and CAI-induced Kyn production (Fig. 2a). CAI strongly induced mRNA and protein expression of a key Try-metabolizing enzyme, IDO1, in both CTLs and B16 tumor tissues (Fig. 2b, c). Kyn can combine with aryl hydrocarbon receptor (AhR) to regulate the expression of many genes. Here, the confocal data indicated that Kyn exposure increased the nuclear import of AhR in CD8^+^ T cells and that this effect was blocked by 3′, 4′-dimethoxyflavone (DMF). To assess whether AhR could increase the expression of PD-1, ChIP-qPCR was performed in CD8^+^ T cells. Our data show that the AhR-dependent expression of PD-1 in activated CD8^+^ T cells in the presence of Kyn tremendously enhanced the activity of the PD-1 transcriptional program (Fig. 2e). The number of PD-1^+^CD8^+^ T cells tended to increase over the time during Kyn treatment. In addition, the combined use of Kyn and DMF resulted in a slight decline but did not counterbalance the percentage of PD-1^+^CD8^+^ T cells (Fig. 2f). To further confirm the role of Kyn in the tumor microenvironment, mice bearing tumors received intratumoral injections of Kyn, DMF or a combination of Kyn and DMF. Similar to the above results, Kyn markedly increased the percentage of PD-1^+^CD8^+^ T cells in TILs and inhibited the production of IFN-γ, while DMF showed a partial offsetting effect, which means that the excess production of Kyn may cause T cell exhaustion and impair the immune surveillance function of CD8^+^ T cells in the tumor microenvironment. These results also suggest that the CAI activation of the IDO-Kyn-AhR cascade might be the underlying mechanism that limits the anti-tumor efficacy of CAI.

### Combining CAI with 1-MT or DMF synergistically disrupts PD-1 expression and promotes IFN-γ production in CD8^+^ T cells

**Fig. 3:**
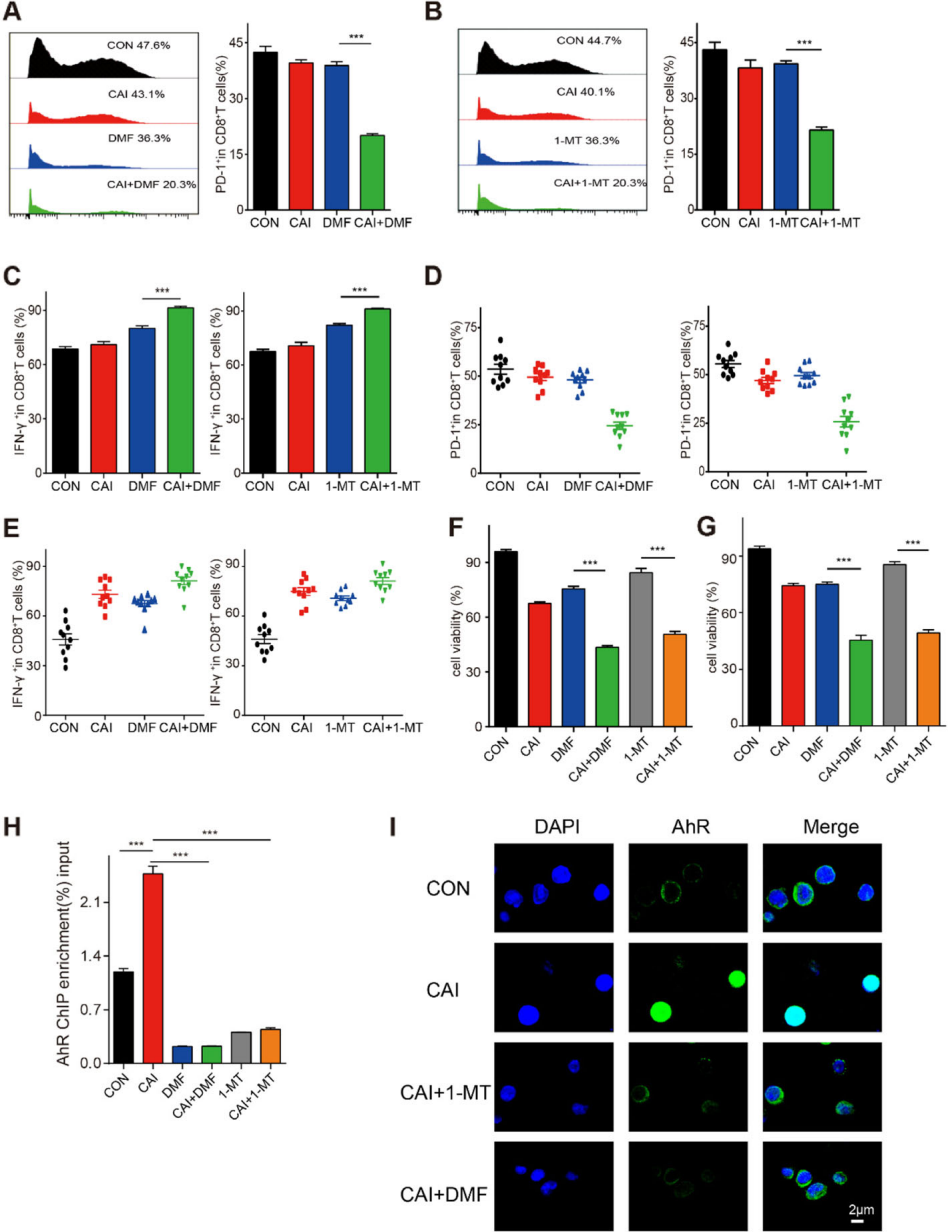
| Combining CAI with 1-MT or DMF synergistically disrupts PD-1 expression and promotes IFN-γ production in CD8^+^ T cells. Sorted CD8^+^ T cells were activated by CD3/CD28 beads for 48 h and treated with CAI (10 μM), DMF (20 μM) or a combination of CAI and DMF for 24 h. In another experiment, the sorted CD8^+^ T cells were treated with CAI (10 μM), 1-MT (0.2 mM) or a combination of CAI and 1-MT for 24 h. **a**-**b** Flow cytometry plots showing the number of PD-1^+^ CD8^+^ T cells after various treatments. Representative histogram plots (left) and the statistical histogram plots (right) are shown. **c** The percentage of IFN-γ-producing T cells in each group was measured by flow cytometry (*n* = 3). **d** and **e** Human peripheral blood mononuclear cells (PBMCs) were isolated from the blood of 10 healthy volunteers, and PBMC-derived CD8^+^ T cells were incubated with the indicated single drug or a combination of drugs for 48 h. The percentages of PD-1-positive cells and IFN-γ-producing cells in each group were measured by flow cytometry. **f** and **g** Activated CD8^+^ T cells from the spleens of C57BL/6 mice were cocultured with B16 cells, or PBMC-derived CD8^+^ T cells were cocultured with HCT116 cells at a ratio of 20:1 for 24 h. Tumor cell apoptosis was analyzed by flow cytometry. **h** ChIP-qPCR analysis of AhR-dependent PD-1 expression after various treatments. The ChIP enrichment ratio relative to the control is shown. **i** CD8^+^ T cells isolated from mouse spleens were activated with anti-CD3/CD28 beads for 48 h. At the same time, activated T cells were treated with CAI (10 μM), DMF (20 μM), 1-MT (0.2 mM) or a combination of CAI and DMF/1-MT for 24 h. Then, the CD8^+^ T cells were fixed and strained with an anti-AhR antibody and imaged by confocal microscopy. Bar, 2 μm. Data are from three independent experiments, and the error bars represent the mean ± s.e.m. **p < 0.01, ***p < 0.001 by one-way ANOVA (A, B, E-H)

To determine whether an IDO1 inhibitor or an AhR inhibitor could improve the effects of CAI on T cells, both mouse spleen-derived CD8^+^ T cells and human PBMC-derived CD8^+^ T cells were treated with CAI, DMF, 1-MT alone or a combination of CAI and DMF or 1-MT for 48 h. There was a statistically significant difference between the monotherapy group and the control group. However, the two combinations drastically decreased PD-1 expression and elevated IFN-γ production in CD8^+^ T cells (Fig. 3a~e). As for human PBMC-derived CD8^+^ T cells, the combination of CAI with DMF or 1-MT also resulted in obvious immune enhancement, as evidenced by the enhancement of PD-1 blockade and an increase in IFN-γ production (Fig. 3d, e). The ChIP-qPCR data showed that CAI facilitated the binding of AhR to the promoter of the PD-1 gene and caused an approximately 2.07-fold increase in PD-1 expression, while combining CAI with DMF or 1-MT obviously reduced the overexpression of PD-1 caused by AhR activation (Fig. 3h). Correspondingly, the immunostaining data showed that the nuclear translocation of AhR was significantly inhibited by the combination of CAI with DMF or 1-MT. (Fig. 3i). The results suggest that the dampening effect of CAI on T cells arising from the activation of the IDO/AhR axis could be overcome by combining CAI with an IDO1/AhR inhibitor and that this combination might play a distinct role in promoting the antitumor immunity of CD8^+^ T cells.

### Combining CAI with DMF or 1-MT increased the number of cytotoxic CD8^+^ tumor-infiltrating T cells and downregulated PD-1 expression

**Fig. 4:**
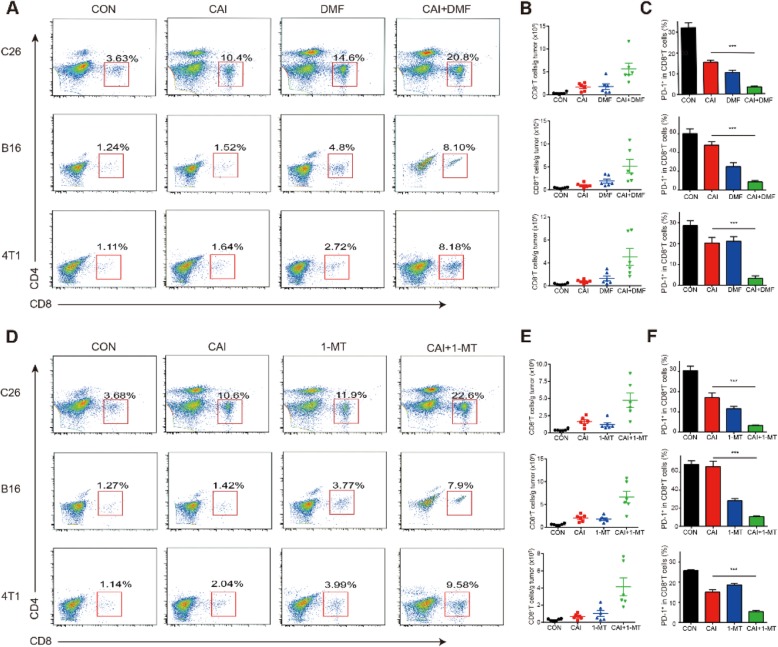
| Combining CAI with DMF or 1-MT increased the number of tumor-infiltrating CD8^+^ T cells and downregulated PD-1 expression. BALB/c or C57BL/6 mice were subcutaneously injected with 1 × 10^6^ tumor cells (C26 cells, B16 cells or 4 T1 cells, *n* = 6 in every group) and received the indicated drugs for 7 days after the day the tumor size reached 5 mm in diameter. T cells in the tumor microenvironment were sorted and analyzed by flow cytometry. **a** Representative flow cytometry plots showing the fraction of CD8^+^ T cells within the CD3^+^ TILs in the C26, B16- or 4 T1- tumor microenvironment. **b** CD8^+^ T cell numbers per gram of tumor in different groups. **c** The percentage of PD-1^+^CD8^+^ T cells within TILs in the tumor microenvironment. **d**, **e** and **f** The same measurements from the evaluation of the effects of another drug combination (CAI and 1-MT) in C26-, B16- or 4 T1- tumor bearing mice. Data are from three independent experiments, and the error bars represent the mean ± s.e.m. **p < 0.01 by one-way ANOVA (**a**-**f**)

To study the effects of CAI, DMF, 1-MT and the two combinations (CAI with DMF/CAI or 1-MT) on T cells within the tumor microenvironment, three tumor-bearing mice models were used. BALB/c or C57BL/6 mice were subcutaneously injected with tumor cells (C26, 4 T1 or B16 cells, *n* = 6 in every group). The mice began to receive drug treatment when the mean size of the tumors reached 5 mm in diameter. Cells were isolated from solid tumor tissues and assayed with flow cytometry. The combination of CAI and DMF showed consistent synergistic effects in the 3 tumor-bearing animal models, which elevated the percentage of CD8^+^ T cells in TILs in all 3 types of tumor tissues five- to eight-fold compared with that in the control group (Fig. 4a, b). Meanwhile, CAI, DMF or a combination significantly reduced the expression of PD-1^+^ in CD8^+^ T cells in the combination group, demonstrating the strengthened inhibitory effect in comparison with that in both monotherapy groups (Fig. 4c). When an alternative inhibitor of IDO1, 1-MT, was used instead of DMF in subsequent independent experiments with the same types of tumor-bearing mice, the abovementioned indicators showed variations consistent with those observed in previous studies (involving DMF) for each monotherapy group and combination group in all 3 types of tumor tissues (Fig. 4d~f). It was speculated that the augmentation of PD-1 blockade might promote CD8^+^ T cell survival and enhance the cytotoxic activity of TILs in the tumor microenvironment. In addition, the effects of the indicated single drugs or combination drugs on other cell types in the tumor microenvironment were comprehensively analyzed by flow cytometry. There were no significant differences in the number and typical function of tumor-associated macrophages (TAMs) between the different treatment groups. The same was true for other cell types, including myeloid-derived suppressor cells (MDSCs), regulatory T cells (Tregs) and CD4^+^ T cells. It is worth mentioning that the downregulation of PD-1 in the combination groups was very obvious in CD8^+^ T cells but not in CD4^+^ T cells (Additional file 3: Figure S3). The results indicate that the enhanced anti-tumor activity of the two combinations of drugs was mainly due to enhanced CD8^+^ T cell function and number.

### Combining CAI with IDO1/AhR inhibitors affected the phenotype and function of transferred T cells in B16-OVA mice and showed beneficial anti-cancer effects

**Fig. 5:**
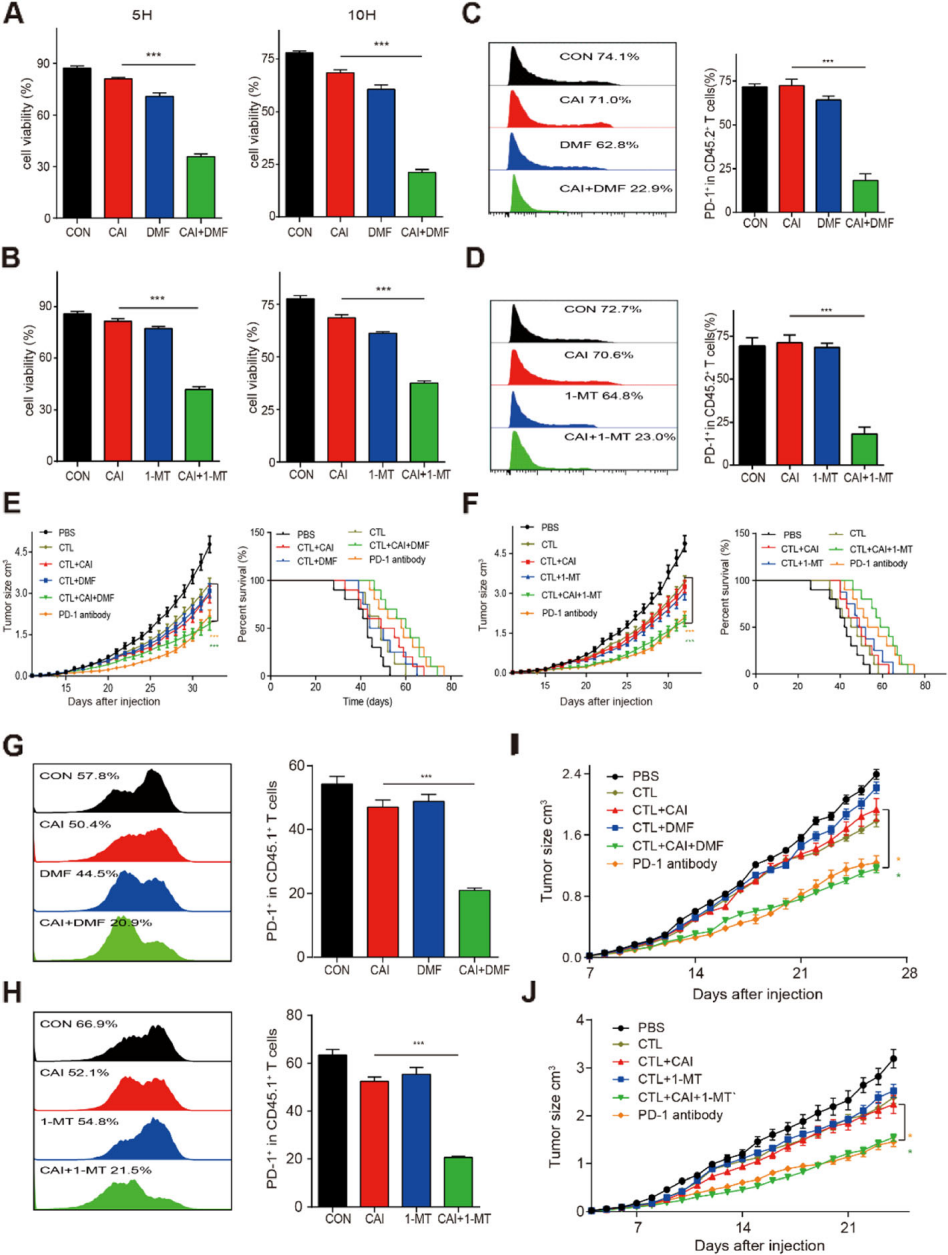
| Phenotypic character of transferred CD45.2 T cells in B16-OVA mice and the in vivo anti-tumor activity of different treatments. **a** and **b**) Activated CD8^+^ T cells from the spleens of OT-1 mice were cocultured with B16-OVA cells at a ratio of 2:1 for 5 h and 10 h and treated with CAI (10 μM), DMF (20 μM) or a combination of CAI and DMF. In another experiment, sorted CD8^+^ T cells were treated with CAI (10 μM), 1-MT (0.2 mM) or a combination of CAI and 1-MT. B16-OVA cell apoptosis was analyzed by flow cytometry at 5 h and 10 h after drug treatment. **c** and **d** CD45.1 C57BL/6 mice bearing 3 × 3 mm OVA-B16 melanomas were subject to the adoptive transfer of OVA-specific CD45.2^+^CD8^+^ T cells (1 × 10^7^ cells/mouse) three times (every 5 days). At the same time, these mice were treated with PBS or CAI (20 mg/kg), 1-MT (5 mg/ml in drinking water), DMF (10 mg/kg), or CAI + 1-MT, CAI + DMF or anti-PD-1 neutralizing antibody (250 μg per mouse) for 20 days. Five days later, several of the mice (*n* = 6) were sacrificed to obtain TILs for flow cytometry, and the remaining mice continued to receive drug treatment. Anti-CD45.2 antibody was used to distinguish donor CD45.2 T cells from host and competitor cells. **c** and **d** Flow cytometry plots showing the expression of PD-1^+^ in CD45.2 ^+^ TILs from B16-OVA CD45.1 mice after various treatments (left: representative histogram plot; right: statistical histogram plot). **e** and **f** Tumor growth was measured (left), and long-term survival was analyzed (right). RAG1 KO mice bearing 3 × 3 mm OVA-B16 melanomas were subject to the adoptive transfer of OVA-specific CD45.1^+^CD8^+^ T cells (1 × 10^7^ cells/mouse) every 5 days. The mice were grouped and administered treatments as indicated above. **g** and **h** Tumors were harvested after 15 days of inoculation, dissociated into single-cell suspensions, and stained for flow cytometry (*n* = 6/group). The expression of PD-1^+^CD45.1^+^ TILs in B16-OVA CD45.2 mice after various treatments was analyzed by flow cytometry (left: representative histogram plot; right: statistical histogram plot). **i** and **j** Tumor growth curves of RAG1 KO mice (*n* = 7/group). Data are from three independent experiments, and the error bars represent the mean ± s.e.m. **p < 0.01, ***p < 0.001 by one-way ANOVA and Kaplan-Meier survival analysis

Combining CAI with IDO1/AhR inhibitors could lead to a more selective anti-tumor immunoreaction, which was confirmed in a specialized coculture system consisting of B16 melanoma cells expressing ovalbumin (OVA) antigen (B16-OVA) and OVA-specific CTLs derived from OT-1 transgenic mice. Either combination resulted in the lowest survival rate of B16-OVA cells in parallel experiments with single agents. (Fig. 5a, b). To further assess the immunotherapeutic effects of the two combinations on T cells, adoptive cell transfer (ACT) was conducted. In terms of the proportion of PD-1^+^CD45.2^+^ TILs in B16-OVA CD45.1 mice, monotherapy caused a slight decrease after 5 days of treatment. Noticeably, combined therapy (CAI + DMF or CAI + 1-MT) resulted in a significant synergistic inhibition compared with either single agent, with the proportion of PD-1^+^CD45.2^+^ TILs decreasing to less than 1/3 of that in the control group (Fig. 5c, d). We then evaluated the anti-tumor effect in vivo using B16-OVA tumor-bearing mice who received T cell ACT. The combination of CAI and DMF and CAI and 1-MT obviously inhibited tumor growth compared with the control group or either single agent-treated group. In addition, prolonged treatment (> 30 days) with the combinations resulted in more encouraging effects that were comparable with those of PD-1 antibody (Fig. 5e, f). We also assessed the anti-tumor activity of the indicated therapeutics in RAG1 KO mice bearing B16-OVA tumors. Each of the three monotherapies, CAI, DMF, or 1-MT, slightly reduced PD-1 expression on the surfaces of transferred CD45.1^+^ T cells and showed little effect on tumor growth. In contrast, combined therapy (CAI + DMF or CAI + 1-MT) significantly reduced the expression of PD-1^+^ in CD45.1^+^ T cells and clearly inhibited tumor growth (Fig. 5g~i).

Interestingly, in RAG1 KO mice bearing B16 tumors, the tumor growth inhibition effect of the indicated treatment (monotherapy or combined therapy) was obviously weaker than that of the same treatment in the presence of transferred CTLs in RAG1 KO mice bearing B16-OVA tumors. Both combinations (CAI and DMF and CAI and 1-MT) had a stronger inhibitory effect on tumor growth than either single drug, but the difference was not significant until the transfer of CTLs was performed (Additional file 4: Figure S4). The results illustrate that T cell-mediated killing plays an important role in the enhanced anti-tumor activity of the two combinations.

### IDO1 or AhR inhibitor enhanced the effect of CAI on xenograft tumors

**Fig. 6:**
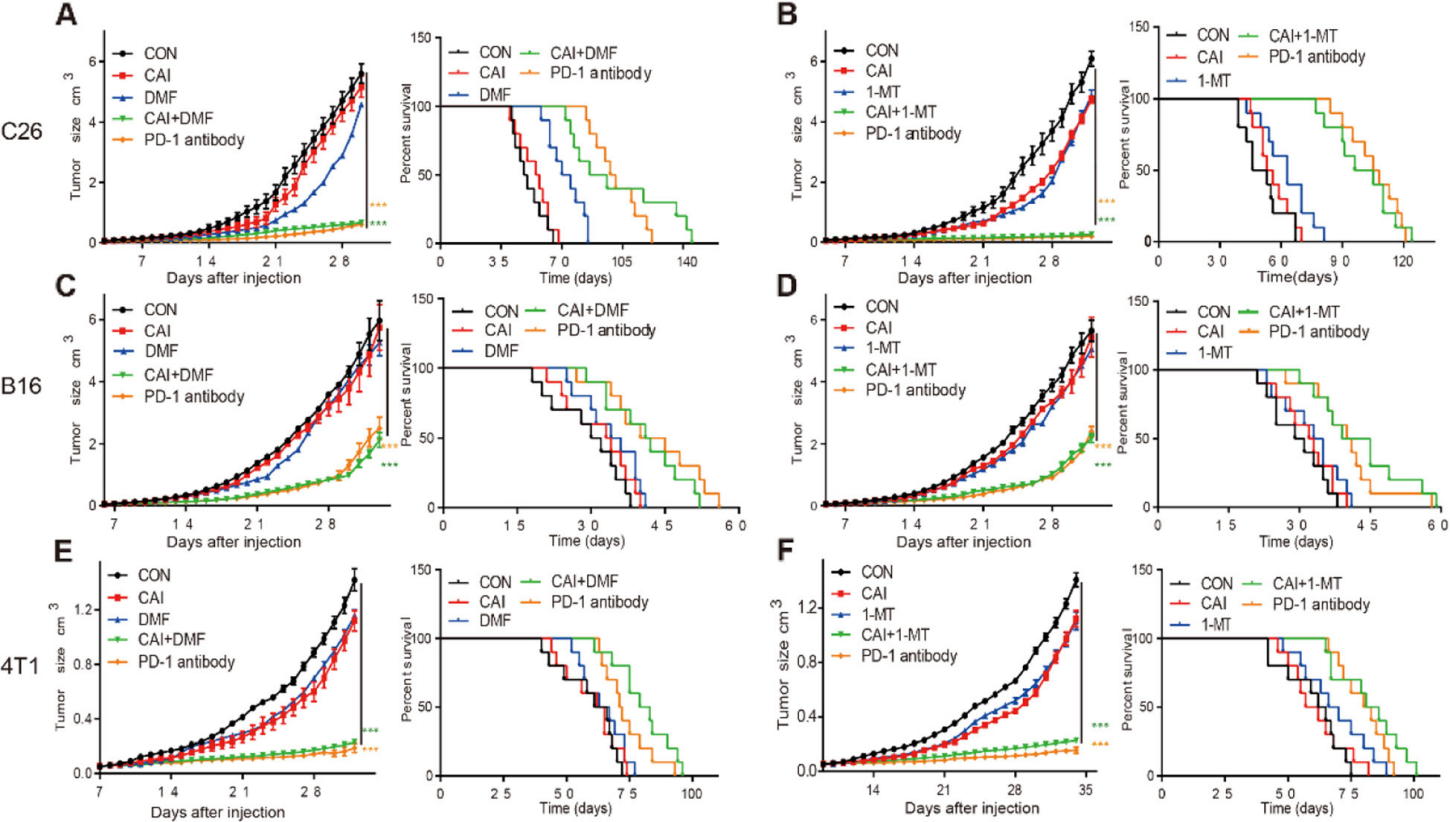
| In vivo anti-tumor effects of CAI, DMF, 1-MT and the combinations of CAI + DMF and CAI + 1-MT with PD-1 antibody as a positive control drug. BALB/c or C57BL/6 mice were subcutaneously injected with B16 (2 × 10^5^ cells/mouse), C26 (1 × 10^5^ cells/mouse) or 4 T1 (1 × 10^5^ cells/mouse) tumor cells. When the tumor size was 5 × 5 mm, the mice were treated with PBS, CAI, 1-MT, CAI/1-MT, and anti-PD-1 neutralizing antibody or PBS, CAI, DMF, CAI/DMF, and anti-PD-1 neutralizing antibody for 28 days. The tumor growth curves and survival curves for tumor-bearing mice (*n* = 10) receiving various treatments are shown as indicated. **a** and **b** C26 colorectal cancer model. **c** and **d** B16 melanoma model. **e** and **f** 4 T1 breast cancer model. The data represent the mean ± s.e.m. ***p < 0.001 by one-way ANOVA (**a**-**f**, left panels) and Kaplan-Meier survival analysis (**a**-**f**, right panels)

Given the important roles of activated T cells in the tumor microenvironment, three types of xenograft tumor models were developed to evaluate the in vivo effects. As shown in Fig. 6, CAI, DMF, or 1-MT alone was able to inhibit tumor growth to a certain extent. The combination of CAI and DMF and CAI and 1-MT led to a dramatic reduction in tumor growth compared with that in the control group or either single agent-treated group in all three types of tumor-bearing mice models, and the anti-tumor effects were comparable to those of anti-PD-1 antibody. Regarding the survival time of tumor-bearing mice, all treatments prolonged the life span of tumor-bearing mice, with the exception that CAI monotherapy provided no improvement in life span in 4 T1 tumor-bearing mice. The capacity of both combined therapeutics to prolong the survival of tumor-bearing mice was similar to or even better than that of anti-PD-1 antibody (Fig. 6a~e). For example, the median survival time of control 4 T1 tumor-bearing mice was approximately 63.5 days. CAI in combination with DMF prolonged the survival time to 81 days, which surpassed that resulting from treatment with the positive control anti-PD-1 antibody (71.5 days) (Fig. 6e). A similar advantage in terms of prolonging survival time was also observed in tumor-bearing mice treated with CAI plus 1-MT.

## Discussion

The authors have been examining the multiple pharmacological effects of the noncytotoxic small molecule compound CAI, which has shown cancer-preventing, anti-angiogenesis and cancer cachexia-fighting properties by inducing cell apoptosis, blocking calcium entry and inhibiting cell oxidative phosphorylation in a variety of studies [22–27]. CAI also increases tumor responses to other anticancer treatments [28]. However, the in vivo anti-tumor activity of CAI and its performance in many clinical trials are barely satisfactory, which prevents it from being a first-line chemotherapy drug.

**Fig. 7:**
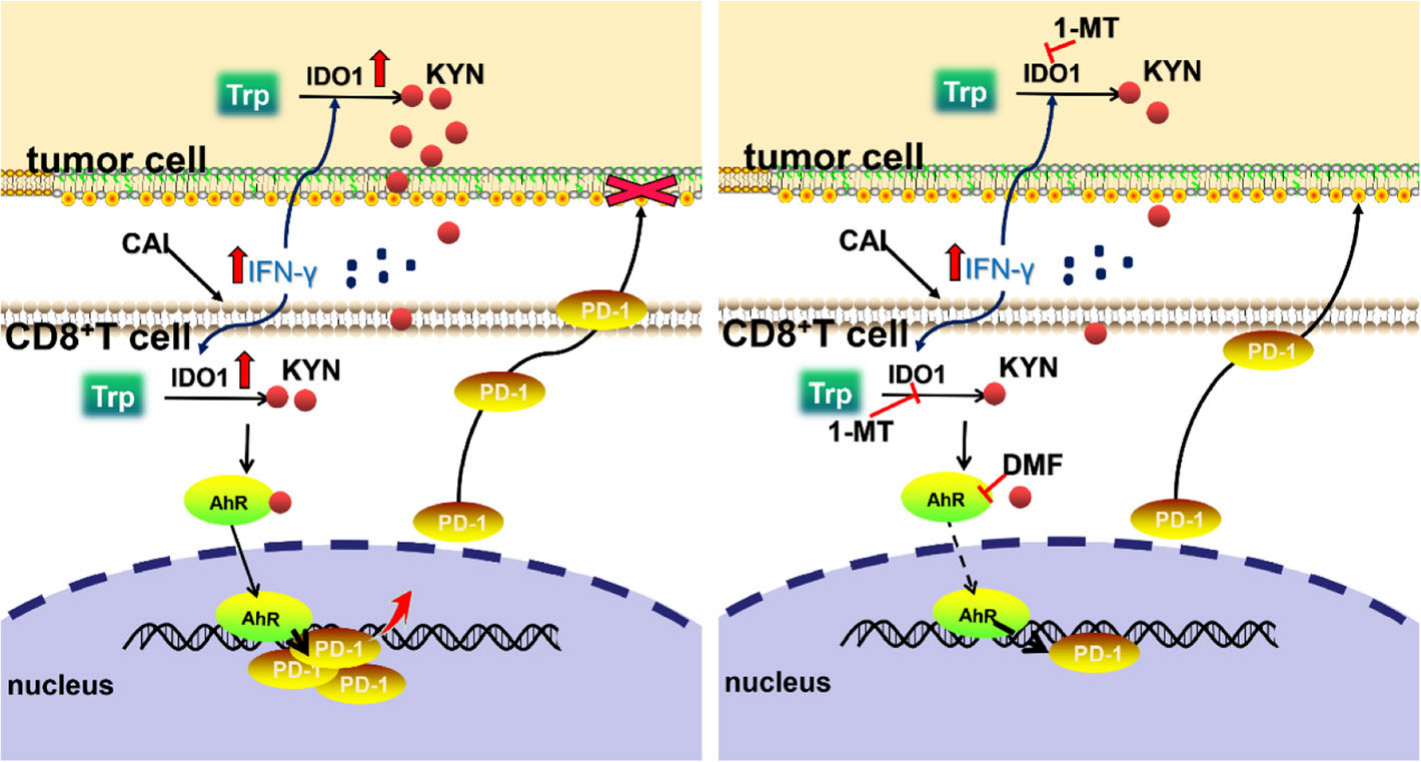
| Schematic diagram illustrating the regulation of the IDO-Kyn-AhR pathway and IFN-γ production in T cells by CAI and the proposed signal modulation mediated by T cell activation

Previously, we focused on synergistically blocking oncogenic signaling pathways in tumor cells and inhibiting cell proliferation with CAI and other combinatorial agents, but we neglected the potential suppressive effect of CAI on immune cells in the tumor microenvironment. Changes in tumor-derived nutrient metabolites in the local microenvironment may affect T cell proliferation and function [29]. For instance, IDO overexpression in tumor cells leads to the depletion of tryptophan, which contributes to an unfavorable environment for T-cell expansion. Instead, tumor-infiltrating lymphocytes produce IFN-γ to suppress tumor cells, and a weakened immune response plays a pivotal role in tumor initiation, growth and metastasis. CAI boosts interferon-γ production by CD8^+^ T cells, which correlates with the abundance and activation of T cells and plays a pivotal role in antitumor host immunity. However, interferon-γ also induces the expression of IDO1, and this might constitute a naturally occurring negative feedback mechanism that regulates the immune response to avoid cross-reactions with normal tissues (Fig. 7).

To abolish the negative effect of CAI on CD8^+^ T cells, 1-MT and DMF were separately combined with CAI, and both of them independently target the upstream and downstream effectors of the IDO-Kyn-AhR-PD-1 pathway (Fig. 7). Although IDO1 is a very important immune checkpoint controller, preclinical studies have noted that single-agent treatment with an IDO1 inhibitor has a negligible effect on decreasing the established cancer burden [30]. Therefore, approaches combining IDO1 inhibitors and other complementary compounds or immune checkpoint inhibitors would probably produce synergistic benefits in terms of tumor growth and animal survival. The present study has confirmed that combining IDO1-Kyn-AhR inhibitors with CAI can greatly augment the activity of CD8^+^ T cells to enhance their killing malignant cells, and the reduced expression of PD-1 and the increase in interferon-γ production in CD8^+^ T cells both play key roles in this (Fig. 7). At the same time, rationally designed small-molecule combinations may also hold promise as adjunctive therapies for patients with other immune suppression-related diseases, such as tuberculosis and HIV.

Anti-PD-1 antibodies have achieved tremendous clinical success in cancer treatment; however, a significant fraction of patients remain unresponsive to these biologic modalities, including CAR-T therapy [31]. Regulating the immune system through alternative pathways with small-molecule compounds may offer complementary benefits when used with biological immunotherapies, including improved feasibility, high oral bioavailability, greater exposure within the tumor microenvironment and lower costs [32]. The two combinations used in the present study were precisely the types of potent approaches with enhanced anti-tumor activity comparable to that of anti-PD-1 antibody that are deserving of further study.

## Conclusion

Inhibitors of the IDO1-Kyn-AhR pathway could abolish the potential negative effects of CAI in the tumor microenvironment. The combination of CAI with 1-MT or DMF greatly augments the activity of CD8^+^ T cells and enhances their killing of malignant cells as a result of the reduced expression of PD-1 and the increase in interferon-γ production. The anti-cancer capacity of the combination of CAI and DMF is superior to that of either single agent and comparable to that of anti-PD-1 antibody, and this is also true for the combination of CAI and 1-MT. The combinations of small molecules introduced in this study may become effective alternate immunotherapy strategies for the treatment of various cancer.

## Additional files

Additional file 1: **Figure S1** | Safety evaluation of drugs. B16 tumor-bearing mice (*n* = 6 in every group) received the indicated drugs for 21 days after the day the tumors reached 5 mm in diameter. (A~E) All mice were sacrificed to detect the levels of blood aminotransferase (ALT), aminotransferase (AST), ALT/AST, urea nitrogen and serum creatinine (Cr E). (F) The tissues shown in the figure were subject to routine HE staining and morphological examination and were observed by a microscope. (DOCX 1420 kb)

Additional file 2: **Figure S2 |** CAI enhanced the anti-tumor activity of CTLs and promoted IFN-γ production. (A) B16 tumor cells and CTLs were cocultured at a ratio of 1:10 or 1:20 for 24 h. The CTLs were preactivated with anti-CD3/CD28 beads in the presence or absence of CAI (10 μM) for 48 h. Tumor cell apoptosis was determined by flow cytometry (left quadrantal diagram), and the tumor cell viability after coculture with CTL is shown in the bar chart. CM: culture medium. (B) HCT116 cells were individually cultured or cocultured with anti-CD3/CD28 bead-activated CTLs at a ratio of 1:10 or 1:20 for 48 h. Then, the cells were treated with vehicle (DMSO) or CAI (10 mM) for 24 h. Tumor cell apoptosis was determined by flow cytometry. (C) Cytokine level changes in the cocultured cell supernatants were detected by ELISA. (D) The interferon content in C26 tumor tissue was detected by ELISA. (DOCX 356 kb) (DOCX 357 kb)

Additional file 3: **Figure S3** | Effects of CAI, CAI + DMF, and CAI + 1-MT on the proportion and typical function of various cell types. Tumors were harvested 14 days after the injection of 2 × 10^5^ C26 cells into BALB/c mice and analyzed by flow cytometry. (**A**) Representative peak plots and statistical histograms showing MHC class-II (two plots on the left) and CD206 expression (two plots on the right) on the surfaces of CD11b-gated TAMs from different groups (*n* = 6). (**B**) Representative (left) or statistical histograms (right) showing the percentage of MDSCs in the tumor microenvironment (*n* = 6). (**C**) Representative (left) or statistical histograms (right) showing the percentage of Tregs within CD45^+^ CD4^+^ cells in the tumor microenvironment (*n* = 6). (**D**) CD4^+^ T cell numbers per gram of tumor in different groups (top). Representative peak plots (middle) and statistical histograms (below) showing the percentage of PD-1^+^CD4^+^ T cells in the tumor microenvironment. (DOCX 513 kb)

Additional file 4: **Figure S4** | CTLs play a great role in the production by CAI + DMF and CAI + 1-MT of enhanced anti-tumor activity. (A) A schematic diagram of tumor inoculation, drug treatment and CTL transfer in RAG1 KO mice. The mice bearing 3 × 3 mm B16 melanomas were treated with PBS, CAI (20 mg/kg), 1-MT (5 mg/ml in drinking water), DMF (10 mg/kg), or CAI + 1-MT, CAI + DMF or anti-PD-1 neutralizing antibody (250 μg per mouse) for 20 days. Ten days after drug administration, the mice began to receive CTL transfers every 5 days (2 times total). (B and C) Tumor growth curves. The arrows indicate the two CTL transfers, which significantly increased the sensitivity of the tumor to combined therapy. (DOCX 228 kb)

## Data Availability

All data are available in this article and the supplementary information files.

## Abbreviations

1-MT: 1-Methyl-L-tryptophan
AhR: Aryl hydrocarbon receptor
CAI: carboxyamidotriazole
CAR-T: Chimeric Antigen Receptor T-Cell Immunotherapy
CTLs: Cytotoxic T lymphocytes
DMF: 3′, 4′-Dimethoxyflavone
IDO1: Indoleamine 2,3-dioxygenase-1
IFN-γ: Interferon-γ
Kyn: Kynurenine
MDSCs: Myeloid-derived suppressor cells
PD-1: Programmed cell death protein 1
PD-L1: Programmed cell death 1 ligand 1
TAM: Tumor-associated macrophages
Tregs: Regulatory T cells
