# Supplementary material for: Carboxyamidotriazole combined with IDO1-Kyn-AhR pathway inhibitors profoundly enhances cancer immunotherapy
Source: J Immunother Cancer. 2019 Sep 11;7:246. doi: 10.1186/s40425-019-0725-7 (PMC6740021; doi:10.1186/s40425-019-0725-7)
Supplement: Supplementary file 1 — Figure S1 | Safety evaluation of drugs. B16 tumor-bearing mice (n = 6 in every group) received the indicated drugs for 21 days after the day the tumors reached 5 mm in diameter. (A~E) All mice were sacrificed to detect the levels of blood aminotransferase (ALT), aminotransferase (AST), ALT/AST, urea nitrogen and serum creatinine (Cr E). (F) The tissues shown in the figure were subject to routine HE staining and morphological examination and were observed by a microscope. (DOCX 1420 kb) [file 40425_2019_725_MOESM1_ESM.docx]

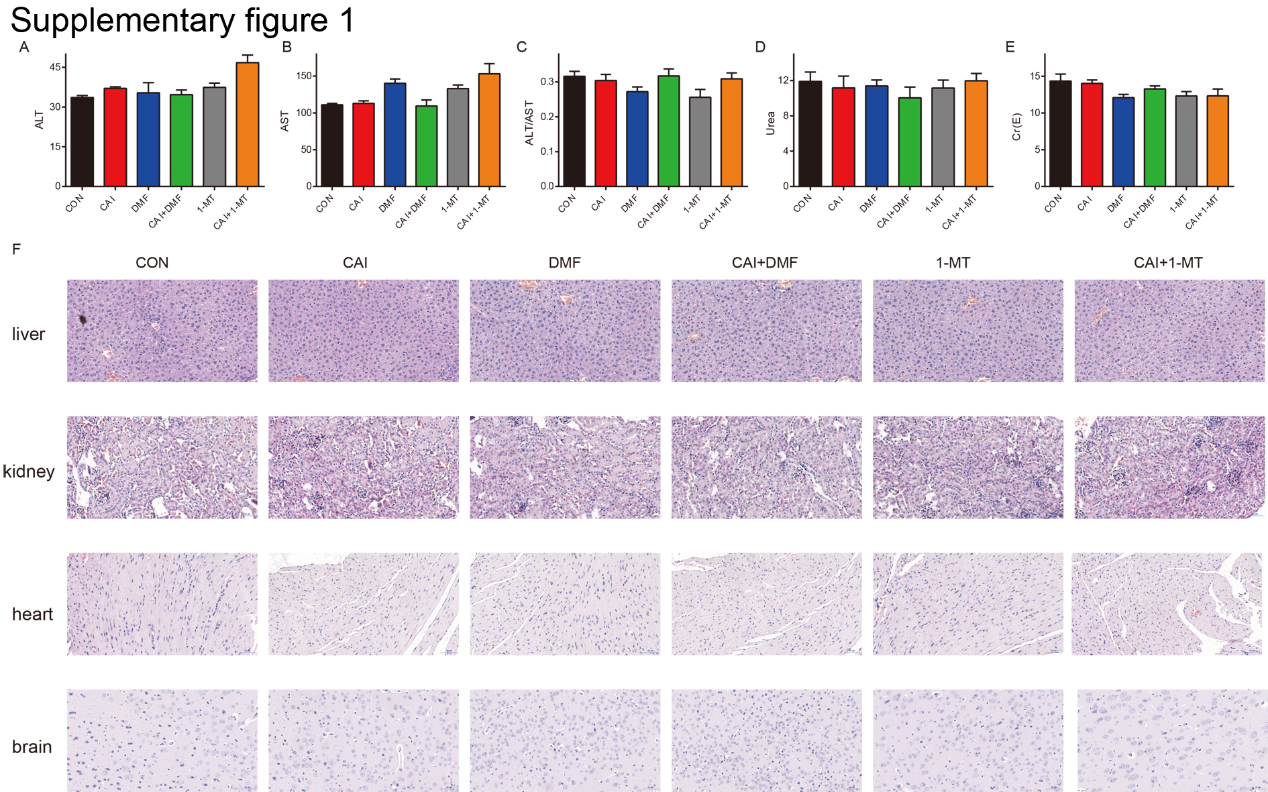


**Additional file 1: Figure S1 | Safety evaluation of drugs.**

B16 tumor-bearing mice (n = 6 in every group) received the indicated drugs for 21 days after the day the tumors reached 5 mm in diameter. **(A~E)** All mice were sacrificed to detect the levels of blood aminotransferase (ALT), aminotransferase (AST), ALT/AST, urea nitrogen and serum creatinine (Cr E). **(F)** The tissues shown in the figure were subject to routine HE staining and morphological examination and were observed by a microscope.
