# Supplementary material for: Carboxyamidotriazole combined with IDO1-Kyn-AhR pathway inhibitors profoundly enhances cancer immunotherapy
Source: J Immunother Cancer. 2019 Sep 11;7:246. doi: 10.1186/s40425-019-0725-7 (PMC6740021; doi:10.1186/s40425-019-0725-7)
Supplement: Supplementary file 2 — Figure S2 | CAI enhanced the anti-tumor activity of CTLs and promoted IFN-γ production. (A) B16 tumor cells and CTLs were cocultured at a ratio of 1:10 or 1:20 for 24 h. The CTLs were preactivated with anti-CD3/CD28 beads in the presence or absence of CAI (10 μM) for 48 h. Tumor cell apoptosis was determined by flow cytometry (left quadrantal diagram), and the tumor cell viability after coculture with CTL is shown in the bar chart. CM: culture medium. (B) HCT116 cells were individually cultured or cocultured with anti-CD3/CD28 bead-activated CTLs at a ratio of 1:10 or 1:20 for 48 h. Then, the cells were treated with vehicle (DMSO) or CAI (10 mM) for 24 h. Tumor cell apoptosis was determined by flow cytometry. (C) Cytokine level changes in the cocultured cell supernatants were detected by ELISA. (D) The interferon content in C26 tumor tissue was detected by ELISA. (DOCX 356 kb) (DOCX 357 kb) [file 40425_2019_725_MOESM2_ESM.docx]

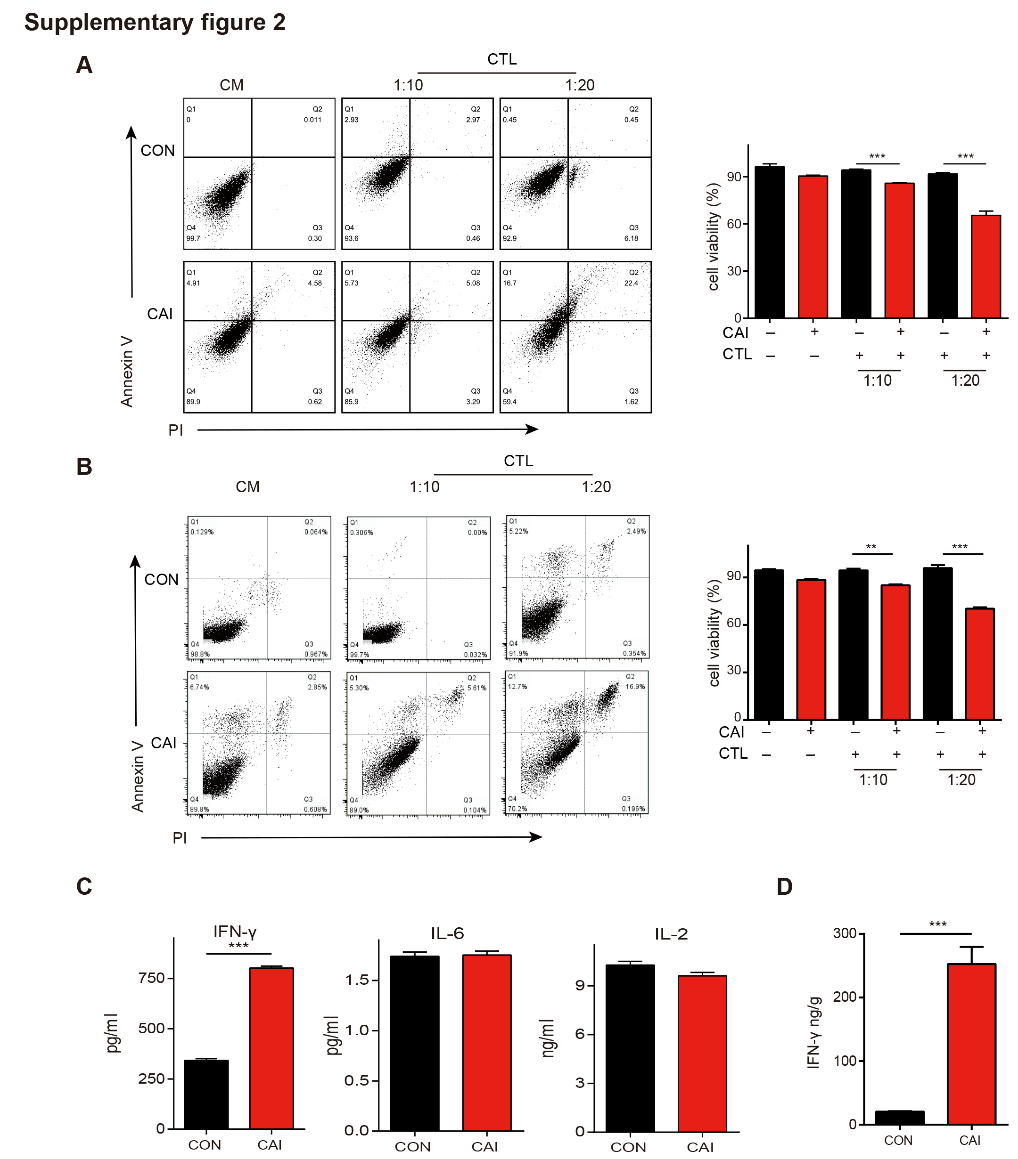


**Additional file 2: Figure S2 | CAI enhanced the anti-tumor activity of CTLs and promoted IFN-γ production.** (A) B16 tumor cells and CTLs were cocultured at a ratio of 1:10 or 1:20 for 24 hr. The CTLs were preactivated with anti-CD3/CD28 beads in the presence or absence of CAI (10 μM) for 48 hr. Tumor cell apoptosis was determined by flow cytometry (left quadrantal diagram), and the tumor cell viability after coculture with CTL is shown in the bar chart. CM: culture medium. (B) HCT116 cells were individually cultured or cocultured with anti-CD3/CD28 bead-activated CTLs at a ratio of 1:10 or 1:20 for 48 hr. Then, the cells were treated with vehicle (DMSO) or CAI (10 mM) for 24 hr. Tumor cell apoptosis was determined by flow cytometry. (C) Cytokine level changes in the cocultured cell supernatants were detected by ELISA. (D) The interferon content in C26 tumor tissue was detected by ELISA.
