# Supplementary material for: Carboxyamidotriazole combined with IDO1-Kyn-AhR pathway inhibitors profoundly enhances cancer immunotherapy
Source: J Immunother Cancer. 2019 Sep 11;7:246. doi: 10.1186/s40425-019-0725-7 (PMC6740021; doi:10.1186/s40425-019-0725-7)
Supplement: Supplementary file 3 — Figure S3 | Effects of CAI, CAI + DMF, and CAI + 1-MT on the proportion and typical function of various cell types. Tumors were harvested 14 days after the injection of 2 × 10^5^ C26 cells into BALB/c mice and analyzed by flow cytometry. (A) Representative peak plots and statistical histograms showing MHC class-II (two plots on the left) and CD206 expression (two plots on the right) on the surfaces of CD11b-gated TAMs from different groups (n = 6). (B) Representative (left) or statistical histograms (right) showing the percentage of MDSCs in the tumor microenvironment (n = 6). (C) Representative (left) or statistical histograms (right) showing the percentage of Tregs within CD45+ CD4+ cells in the tumor microenvironment (n = 6). (D) CD4+ T cell numbers per gram of tumor in different groups (top). Representative peak plots (middle) and statistical histograms (below) showing the percentage of PD-1+CD4+ T cells in the tumor microenvironment. (DOCX 513 kb) [file 40425_2019_725_MOESM3_ESM.docx]

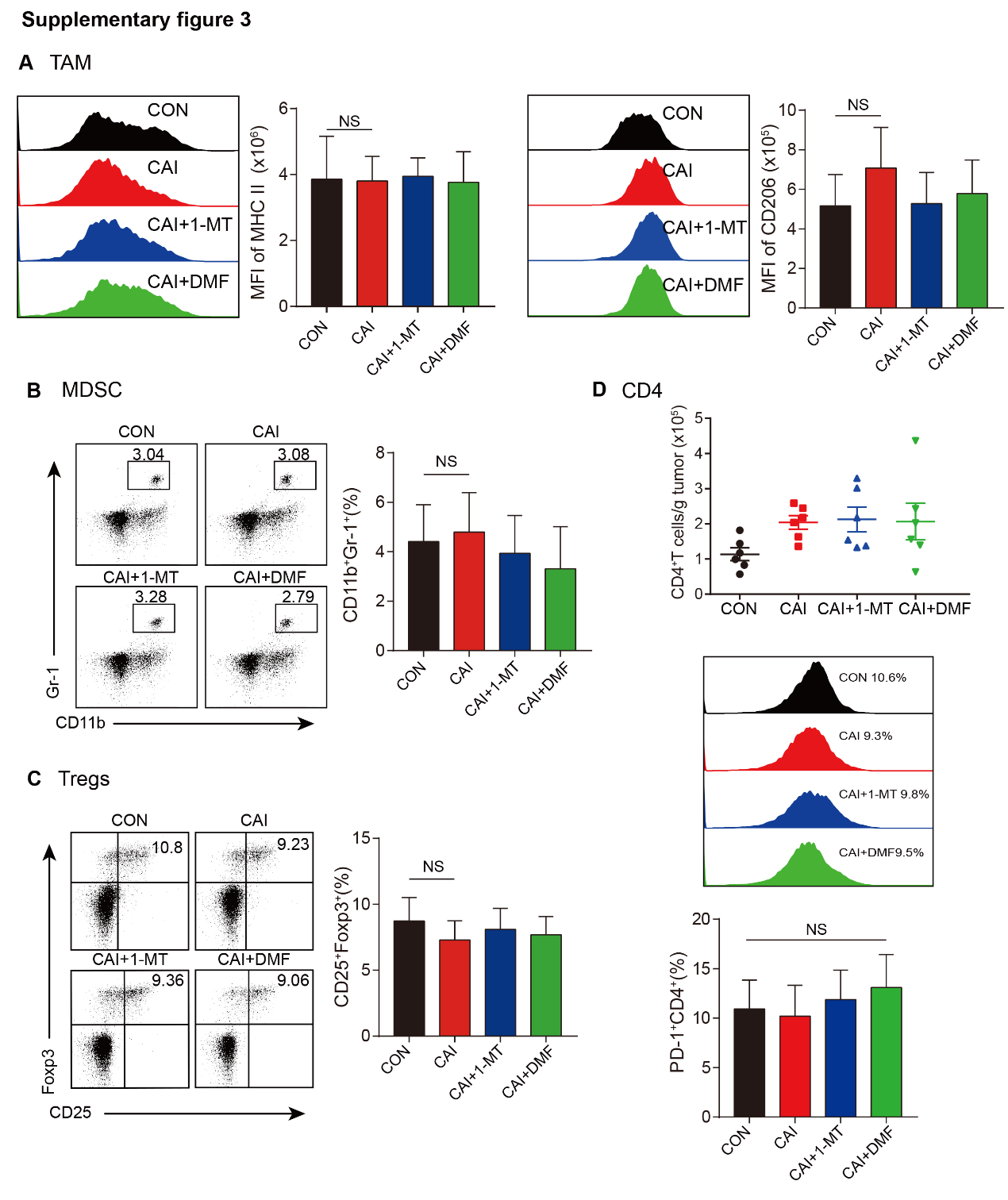
**Additional file 3: Figure S3 | Effects of CAI, CAI+DMF, and CAI+1-MT on the proportion and typical function of various cell types**

Tumors were harvested 14 days after the injection of 2×10^5^ C26 cells into BALB/c mice and analyzed by flow cytometry. **(A)** Representative peak plots and statistical histograms showing MHC class-II (two plots on the left) and CD206 expression (two plots on the right) on the surfaces of CD11b-gated TAMs from different groups (n = 6). **(B)** Representative (left) or statistical histograms (right) showing the percentage of MDSCs in the tumor microenvironment (n = 6). **(C)** Representative (left) or statistical histograms (right) showing the percentage of Tregs within CD45^+^ CD4^+^ cells in the tumor microenvironment (n = 6). **(D)** CD4^+^ T cell numbers per gram of tumor in different groups (top). Representative peak plots (middle) and statistical histograms (below) showing the percentage of PD-1^+^CD4^+^ T cells in the tumor microenvironment.
