# Supplementary material for: Carboxyamidotriazole combined with IDO1-Kyn-AhR pathway inhibitors profoundly enhances cancer immunotherapy
Source: J Immunother Cancer. 2019 Sep 11;7:246. doi: 10.1186/s40425-019-0725-7 (PMC6740021; doi:10.1186/s40425-019-0725-7)
Supplement: Supplementary file 4 — Figure S4 | CTLs play a great role in the production by CAI + DMF and CAI + 1-MT of enhanced anti-tumor activity. (A) A schematic diagram of tumor inoculation, drug treatment and CTL transfer in RAG1 KO mice. The mice bearing 3 × 3 mm B16 melanomas were treated with PBS, CAI (20 mg/kg), 1-MT (5 mg/ml in drinking water), DMF (10 mg/kg), or CAI + 1-MT, CAI + DMF or anti-PD-1 neutralizing antibody (250 μg per mouse) for 20 days. Ten days after drug administration, the mice began to receive CTL transfers every 5 days (2 times total). (B and C) Tumor growth curves. The arrows indicate the two CTL transfers, which significantly increased the sensitivity of the tumor to combined therapy. (DOCX 228 kb) [file 40425_2019_725_MOESM4_ESM.docx]

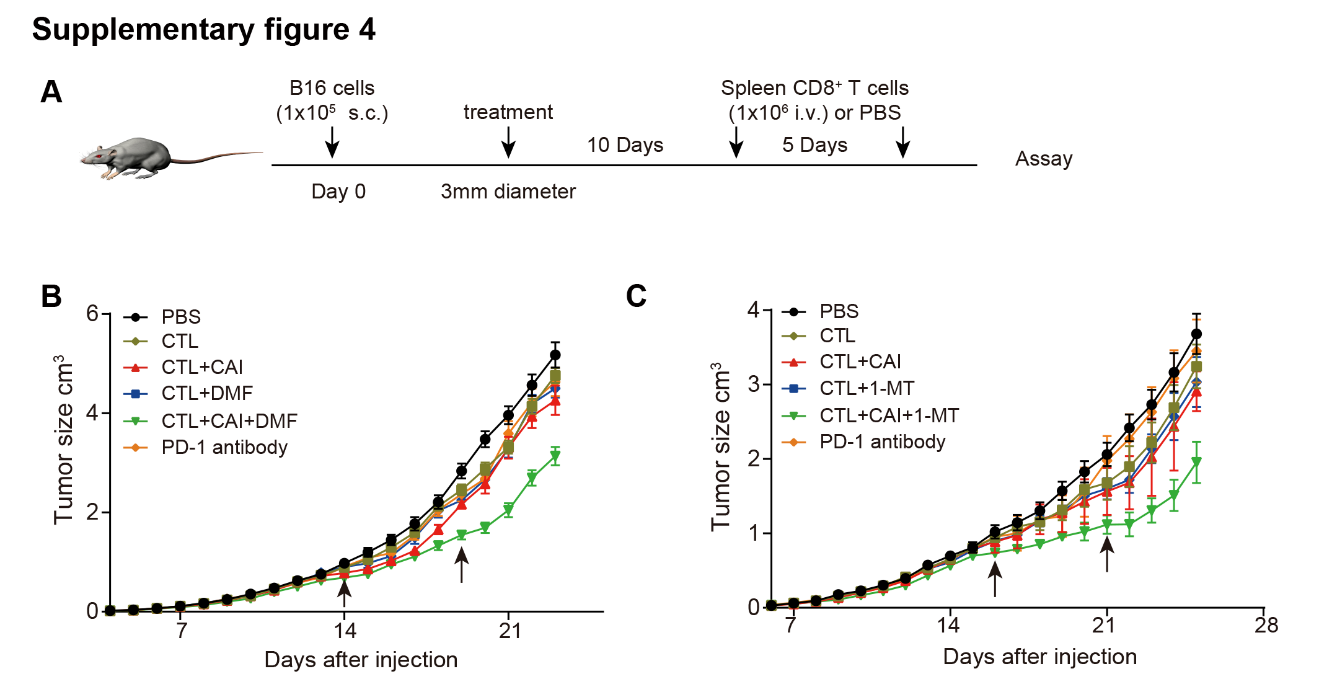
**Additional file 4: Figure S4 | CTLs play a great role in the production by CAI+DMF and CAI + 1-MT of enhanced anti-tumor activity.**

(A) A schematic diagram of tumor inoculation, drug treatment and CTL transfer in RAG1 KO mice. The mice bearing 3×3 mm B16 melanomas were treated with PBS, CAI (20 mg/kg), 1-MT (5 mg/ml in drinking water), DMF (10 mg/kg), or CAI+1-MT, CAI+DMF or anti-PD-1 neutralizing antibody (250 μg per mouse) for 20 days. Ten days after drug administration, the mice began to receive CTL transfers every 5 days (2 times total). (B and C) Tumor growth curves. The arrows indicate the two CTL transfers, which significantly increased the sensitivity of the tumor to combined therapy.
